# Microsatellites grant more stable flanking genes

**DOI:** 10.1186/1756-0500-5-556

**Published:** 2012-10-05

**Authors:** Reem Joukhadar, Abdulqader Jighly

**Affiliations:** 1 University of Aleppo, Aleppo, Syria; 2 International Center for Agricultural Research in the Dry Areas (ICARDA), P.O. Box 5466, Aleppo, Syria

**Keywords:** Microsatellite, Polymerase Chain Reaction (PCR), Polymerase slippage, Transformation

## Abstract

**Background:**

Microsatellites, or simple sequence repeats (SSRs), are DNA sequences that include tandem copies of specific sequences no longer than six bases. SSRs are ubiquitous in all genomes and highly mutable.

**Presentation of the hypothesis:**

Results from previous studies suggest that flanking regions of SSR are exhibit high stability in a wide range of organisms. We hypothesized that the SSRs ability to discard weak DNA polymerases could be responsible for this unusual stability. . When the weak polymerases are being decayed over SSRs, the flanking sequences would have higher opportunity to be replicated by more stable DNA polymerases. We present evidence of the molecular basis of our hypothesis.

**Testing the hypothesis:**

The hypothesis could be tested by examining the activity of DNA polymerase during and after a number of PCRs. The PCR reactions should be run with the same SSR locus possessing differences in the SSR length. The hypothesis could also be tested by comparing the mutational rate of a transferred gene between two transformations. The first one has a naked T-DNA (transferred DNA), while the second one has the same T-DNA flanked with two SSRs.

**Implications of the hypothesis:**

In any transformation experiment, flanking the T-DNA fragment with SSR sequences would result in more stably transferred genes. This process would decrease the unpredictable risks that may occur because of the mutational pressure on this foreign segment.

## Background

Microsatellite or simple sequence repeats (SSRs) are tandem repeated DNA sequences including tandem copies of specific sequences no longer than six bases [1]. SSRs are ubiquitously distributed almost in all eukaryotic and prokaryotic genomes [2]. Their abundance and the tandem repeated nature of SSRs make the loci highly mutable loci. Two mechanisms have been attributed to this phenomenon namely [3] unequal crossing over [4] and slipping of the DNA polymerase when it run over the SSR template strand allowing one strand to hybridize with one of the multi-complimentary tandem sequences on the other strand [5]. These mechanisms results in SSR loci being highly mutable [6]; this unique mutational ratio creates a need to align these loci in a special way [7].

There are a lot of factors that affect the mutational ratio. The longer SSRs are more variable than shorter ones; however there is no threshold for slippage occurrence [8]. It appears that SSR mutations are dependent on motif size and nucleotide content. Besides, position in genome (coding or non-coding sequences), presence on leading or lagging strand and the distance from origin of replication also affect SSR mutations [9]. Further, fidelity of replication and repair mechanisms as well as epigenetic factors may influence repeat stability [9].

Despite the high mutation rate of SSRs, their flanking regions exhibit high stability even among different taxa; there is a negative correlation between SSR length and substitution rate in nearby flanking sequence [10,11]. SSRs are increasingly being used as genetic markers for a wide range of applications such as evolution and diversity studies, genetic mapping and forensic studies [12].

## Presentation of the hypothesis

Our new hypothesis assumes that SSRs may have the power to discard weak DNA polymerases and keep the more robust ones. This selective capacity of SSRs can lead to more accurate amplification of the microsatellite flanking regions. Further, SSR slippage may occur during the decaying and the replacment of weak polymerases. The following paragraphs would present a review on the stability of SSR flanking regions in many organisms and some supportive evidence- related to the conditions of the polymerization as well as our new molecular view of how the selection is done.

### The stability of SSR flanking regions

The stability of the SSR flanking regions was investigated in different organisms; three *Phytophthora* species (algae) [13]; rice and bamboo [14]; cotton [15]; *Brassica rapa* and *Arabidopsis thaliana*[16]; cowpea, mung bean and adzuki bean [17]; *Castanea* spp. [18]; Carya [19] and wheat [20]. Similar studies have also been carried out in different animals such as fish [21]; Acropora [22]; cattle, sheep, yak, buffalo and goat [23]; *Probarbus jullienii*[24] and gerbils [25]; and in human genome studies where different SSR's flanking regions associated with genes expressed in the developing nervous system were compared [26]. In addition, flanking regions of EST-SSR loci demonstrated a common evolutionary origin of grass fungal endophytes taxa [27].

Recently results with pepper chloroplast genome showed that there are two sites called inverted repeat (IRa and IRb) containing high frequency of tandem repeats [28]. In this study, most of the hotspot regions in pepper chloroplast genome seem to be relayed at the middle of the biggest fragment LSC (large single copy) –about 87 kb– which is far from the IR sites.

These studies provide indication that SSRs may help to protect their flanking regions from different kinds of mutations.

### Evidences related to polymerization conditions

Viguera *et al.*[29] reported that the DNA polymerase dissociates from the synthesize strand when the slippage occurs. We think that this dissociation support our assumption when SSRs replace weak DNA polymerases.

It has been reported that SSRs found on the lagging strand shows higher instability than those on the leading strand without any explanation [30]. This lends credence to our assumption because of the presence of Okazaki fragments when lagging strand is being replicated. Replicating those fragments would allow more DNA polymerases to act on them leading to a higher opportunity for weaker DNA polymerases to replicate on the lagging strand.

Further, a significant increase in DNA polymerase slippage rates was reported as a result of inefficient concentrations of ions (particularly Mg ions) during DNA replication [29,31]. It has been reported that magnesium-induced the assembly of a complete DNA polymerase catalytic complex [32].

Kumar *et al.*[33] reported that the misbalancing of dNTPs increases the mutagenesis ratio. Consistent with our hypothesis, we suggest that the long motifs with one or two types of nucleotides could cause more decayed DNA polymerases because they may create a local misbalancing of dNTPs by using limited types of nucleotides which may enhance mutation occurrences.

### Molecular view

**Figure 1:**
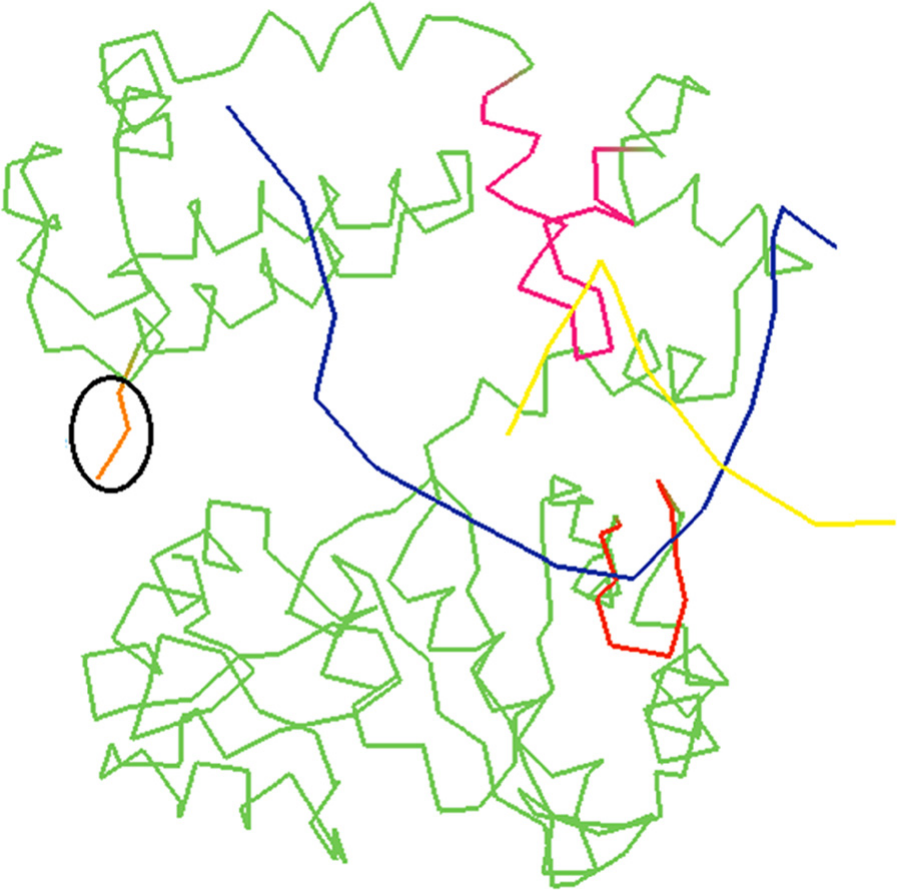
**Ribbon diagram of DNA polymerase (PDB code 1BPX - Sawaya *et al. *[ **[30]**]).** The yellow line is the synthetic DNA strand. The blue line is the template DNA strand. The pink and red subunits are the bracket-like proteomic subunits that join the DNA strands together. The orange line is the free end of the DNA polymerase.

**Figure 2:**
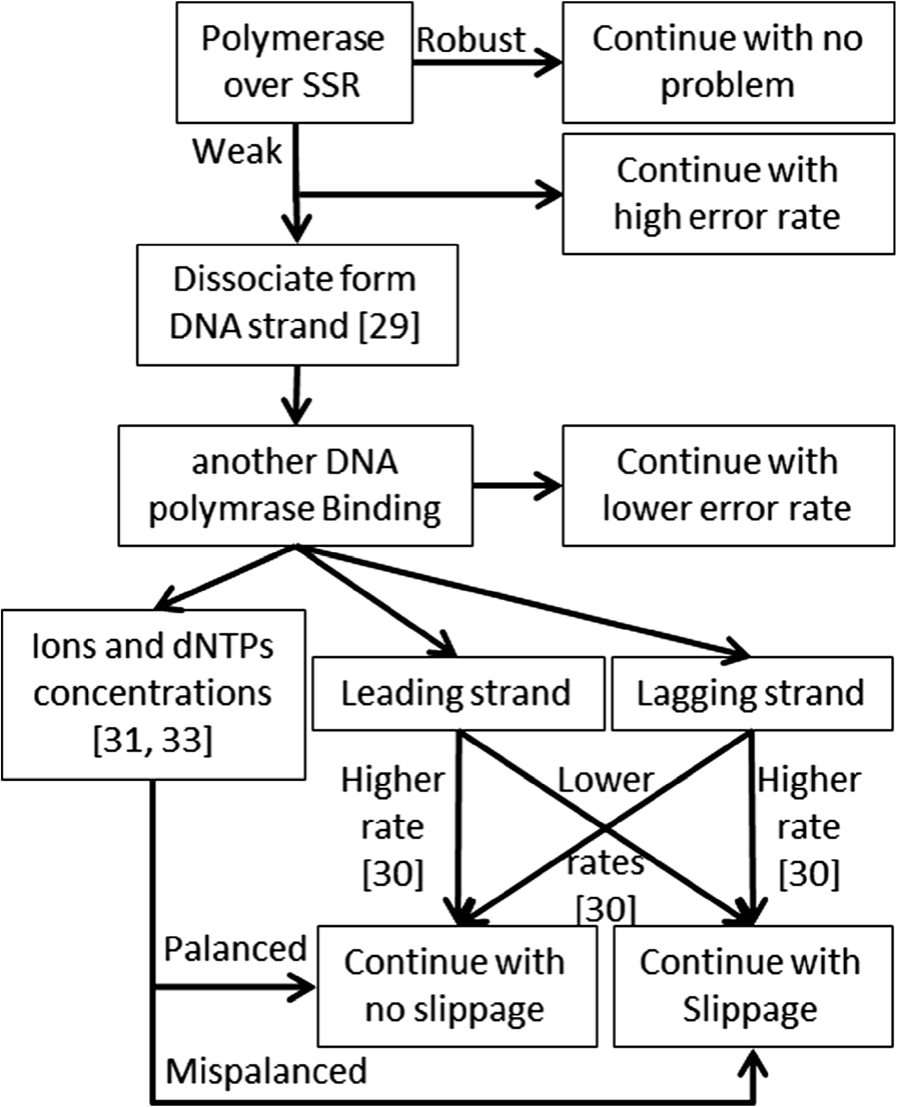
Diagram explains the mechanism of the suggested hypothesis.

Figure 1 illustrates a ribbon diagram of one of the DNA polymerases (PDB code - 1BPX) [34]. As observed, both the template (blue) and the synthetic strands (yellow) are joined with bracket-like proteomic subunit colored with red and pink. This subunit forms a pipe that fit the 3D structure of the double stranded DNA [35,36]. The structure of this subunit prevents the DNA from formation of possible secondary structures (like the loop that occurs when the DNA polymerase slips) [36,37]. While this subunit is stable, the whole protein would be stable and the synthetic strand would have less errors. After building a huge number of bases, the instability of the DNA polymerase would increase as well as the errors rate [38]. Therefore, when the weak polymerase gets over an SSR locus, it would create a suitable environment to generate such loop because of the multi-complementary sequences. In vivo, the DNA polymerase would stop releasing double stranded DNA [29]. Further, the helicase enzyme would still be pushing unwinded strand toward the DNA polymerase [39-41]. This would bring heavy pressure on the weak polymerase. We assumed that when polymerase slips, its domains would separate and the free end would split from the other domain causing a decayed protein and the polymerase would lose its polymerization activity. It's also observed that this slippage happens in vitro during PCR reaction [5,31] and it has previously been reported that repeats undergo deletion if replication temperature is high [42]. Figure 2 demonstrates a diagram describing the whole process.

## Testing the hypothesis and implications of the hypothesis

When trying to improve genetic modified organisms, it would be helpful to add an SSR region to the T-DNA. Flanking the transferred gene with SSRs on both sides would decrease the unpredictable risks that may occur if the foreign T-DNA fragment mutates. Microsatellites have been used in the T-DNA as a molecular marker in order to select the transgenic cucumber lines [43]. Microsatellites of T-DNA showed high differences in their stability. For instance, the CAG repeat of the Huntington disease exposes high stability in transgenic mice [44]. Another study showed an instable transferred microsatellite [45]. However, none of these studies or other publications that used transgenic organisms focused their studies on the stability of microsatellite flanking regions.

Our assumption could be tested by comparing the mutation rate of a transferred gene between two cases - the control and testing cases. The first is the control case, which has the T-DNA, while the second, the testing case, has the same T-DNA flanked by two SSRs. If there were significant differences between both cases, the hypothesis might be applicable. The hypothesis could also be tested by checking the DNA polymerase activities during and after many PCRs. All reactions should be carried out for the same SSR locus with differences in the SSR length and another reaction should be done without the SSR itself.

## Competing interests

The authors declare that they have no competing interests.

## Authors’ contributions

AJ and RJ discussed the hypothesis, drafted and approved the final manuscript.
